# New asymptomatic thrombosis caused by carotid web during the acute period of cerebral infarction

**DOI:** 10.1186/s12883-023-03316-1

**Published:** 2023-07-12

**Authors:** Yan Wang, Hai-Lei Li, Xiao-Hua Xu, Jin-Hao Ye, Jian Li

**Affiliations:** 1 Department of Ultrasound, The University of Hong Kong-Shenzhen Hospital, No.1, Haiyuan Road, Futian District, Shenzhen, 518053 China; 2 Division of Vascular Surgery, Department of Surgery, The University of Hong Kong-Shenzhen Hospital, No.1, Haiyuan Road, Futian District, Shenzhen, 518053 China; 3 Department of Neurology, The University of Hong Kong-Shenzhen Hospital, No.1, Haiyuan Road, Futian District, Shenzhen, 518053 China; 4 Department of Radiology, The University of Hong Kong-Shenzhen Hospital, No.1, Haiyuan Road, Futian District, Shenzhen, 518053 China

**Keywords:** Carotid web, Asymptomatic thrombosis, Cerebral infarction, Carotid ultrasound, Carotid endarterectomy

## Abstract

**Background:**

At present, the carotid web (CaW) as an important cause of cryptogenic ischemic stroke has gradually received clinical attention. CaW is associated with a high risk of stroke and patient is more likely to have recurrent stroke if the CaW is untreated. We report a patient who developed CaW related thrombosis during the acute period of cerebral infarction.

**Case presentation:**

A 49-year-old male patient with CaW in the left internal carotid artery was diagnosed by computed tomography angiography (CTA) and had two cerebral infarctions in two years. Within 72 h after thrombolysis for an acute cerebral infarction, acute thrombosis was identified between the web and the posterior wall of the carotid artery on carotid ultrasound. Emergent carotid endarterectomy (CEA) was performed to remove abnormal CaW structures and thrombosis to prevent stroke. The patient recovered well and was asymptomatic at 2 months follow-up.

**Conclusion:**

Carotid web related thromboembolism is a rare cause of stroke. Carotid ultrasound plays an important role in the diagnosis of asymptomatic thrombosis caused by carotid web. Carotid endarterectomy is effective for stroke prevention in patient with carotid web related thrombosis.

## Background

As a rare cause of acute cerebral infarction, carotid web (CaW) has attracted clinical attention since decades ago [1]. The histological definition of CaW is atypical fibromyodysplasia, the spine-like luminal protrusion of CaW is an important pathological basis for thrombosis [2]. Most CaWs are associated with a high risk of stroke, leading to recurrent symptoms of cerebral ischemia and infarction [1, 3].

Duplex ultrasound and computed tomography angiography (CTA) are recommended as the diagnostic imaging for CaW [2]. The characteristic of CaW on CTA is a shelf like filling defect protruding from the lumen of the carotid artery in the sagittal plane, and a linear defect of lumen division appears in the axial view, usually at the origin of the internal carotid artery (ICA), and most of them originate from the posterior lateral wall of the proximal ICA [4]. CaW was found in 2.5% of patients with large vessel occlusive acute ischemic stroke by CTA examination, most of them were female [5]. In this study, we report a patient with CaW associated stroke who developed asymptomatic thrombosis again in the acute period of cerebral infarction (within 72 h).

### Case presentation

**Fig. 1:**
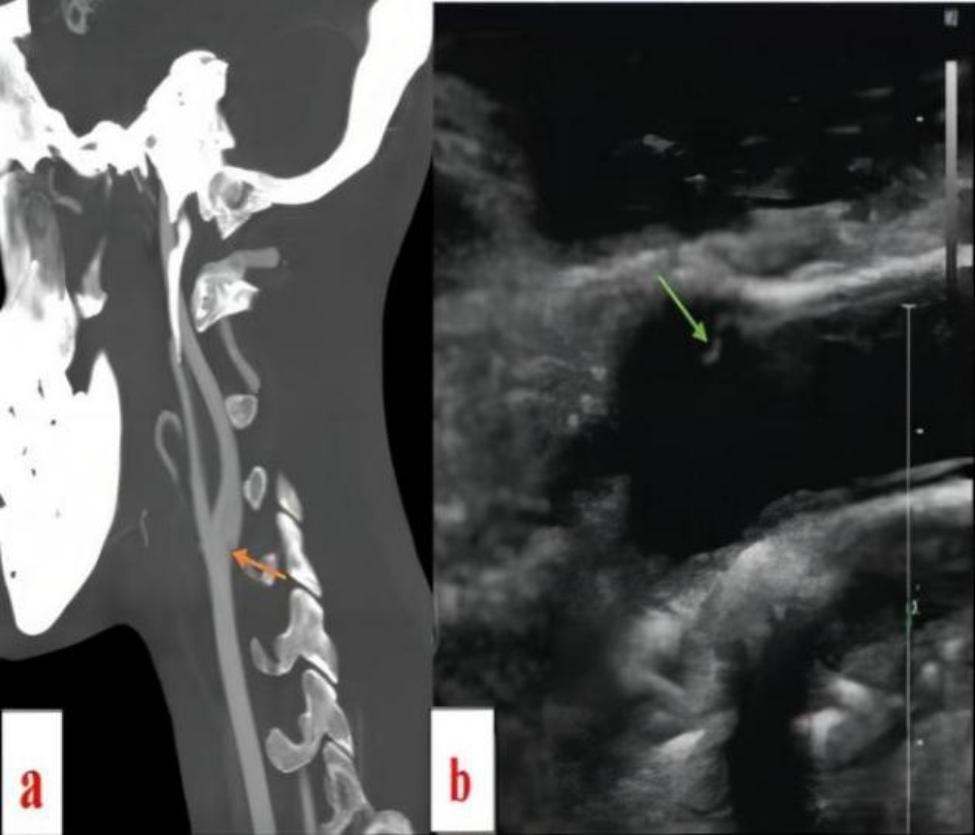
**a** CTA showed CaW (orange arrow), **b** Carotid ultrasound showed CaW (green arrow)

A 49-year-old male presented with aphasia and right limb weakness for four hours. He had a past medical history of stroke, and Caw had been identified on CTA (Fig. 1a) and carotid ultrasound (Fig. 1b) during previous hospitalization. He denied a past medical history of hypertension, diabetes or coronary arterial disease. Since the first ischemic stroke, the patient had been taking anti-platelet drugs and statins continuously to prevent recurrence of stroke .

**Fig. 2:**
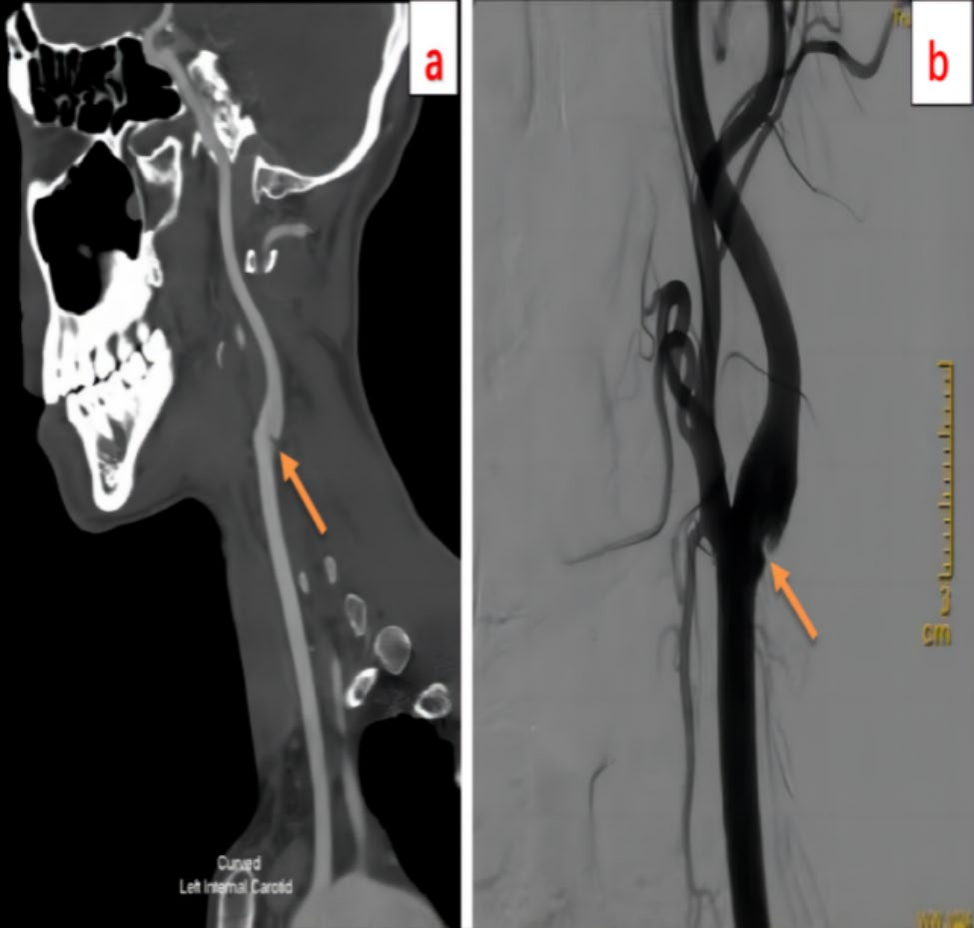
**a**,**b** CTA and DSA showed CaW (orange arrow), no filling defect

On physical examination, he was unconscious, somnolence, aphasia. He had right-sided central facio-lingual palsy, and right limb muscle strength was grade 4 (Lovett scale). The National Institute of Health stroke scale (NIHSS) score was 8, and Glasgow Coma Scale (GCS) score was 4/2/6. Blood cell and coagulation tests were done in Accident and Emergency Department and the results were unremarkable. Head CT scan showed no significant abnormalities. Acute cerebral infarction was diagnosed based on the symptoms and intravenous thrombolytic therapy with rt-PA (0.9 mg/Kg) was given. The patient recovered well after thrombolysis, and the NIHSS score was improved to 1. About 30 min after thrombolysis, urgent CTA of head and neck was done and linear filling defect was identified at the origin of left internal carotid artery (ICA) (Fig. 2a). The distal M1 segment of left middle cerebral artery (MCA) was close to complete occlusion. Cerebral CT perfusion (CTP) showed the perfusion was delayed in the left cerebral hemisphere: mean transit time (MTT) and time to peak (TTP) were prolonged, cerebral blood flow (CBF) and cerebral blood volume (CBV) were slightly raised. About 75 min after thrombolysis, emergency cerebral digital subtraction angiography (DSA) was performed. During intraoperative angiography, we found the occluded segment of MCA was successfully recanalized, so cerebral artery thrombectomy was not performed. CaW was observed at the origin of the left internal carotid artery, contrast retention without filling defect was observed at the carotid bulb (Fig. 2b). After 24 h of thrombolysis, repeated CTP showed that the abnormal perfusion area was significantly reduced and was limited to the left basal ganglia. The patient was treated with aspirin, and his condition was stable. During this period, the patient’s blood pressure was maintained at about 100/70mmHg for a long time without any antihypertensive drugs.

**Fig. 3:**
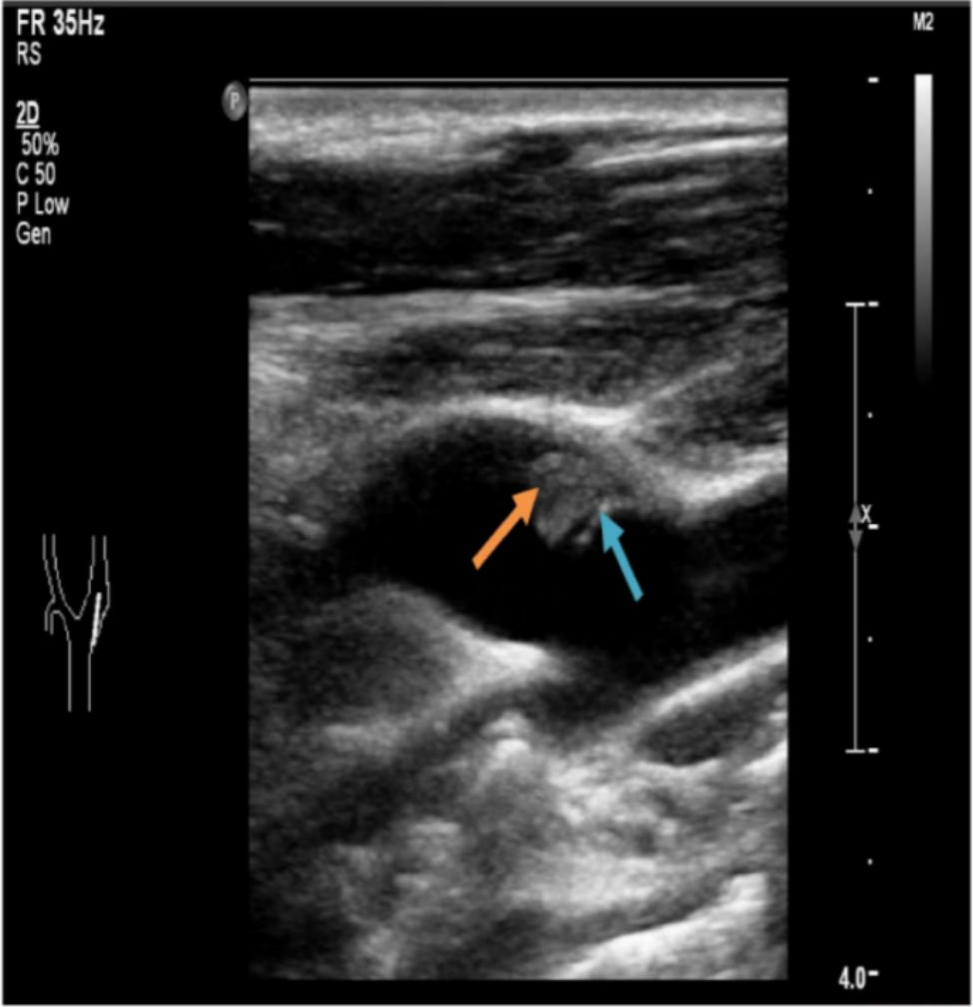
Carotid ultrasound showed CaW (blue arrow) and a new thrombus (orange arrow)

**Fig. 4:**
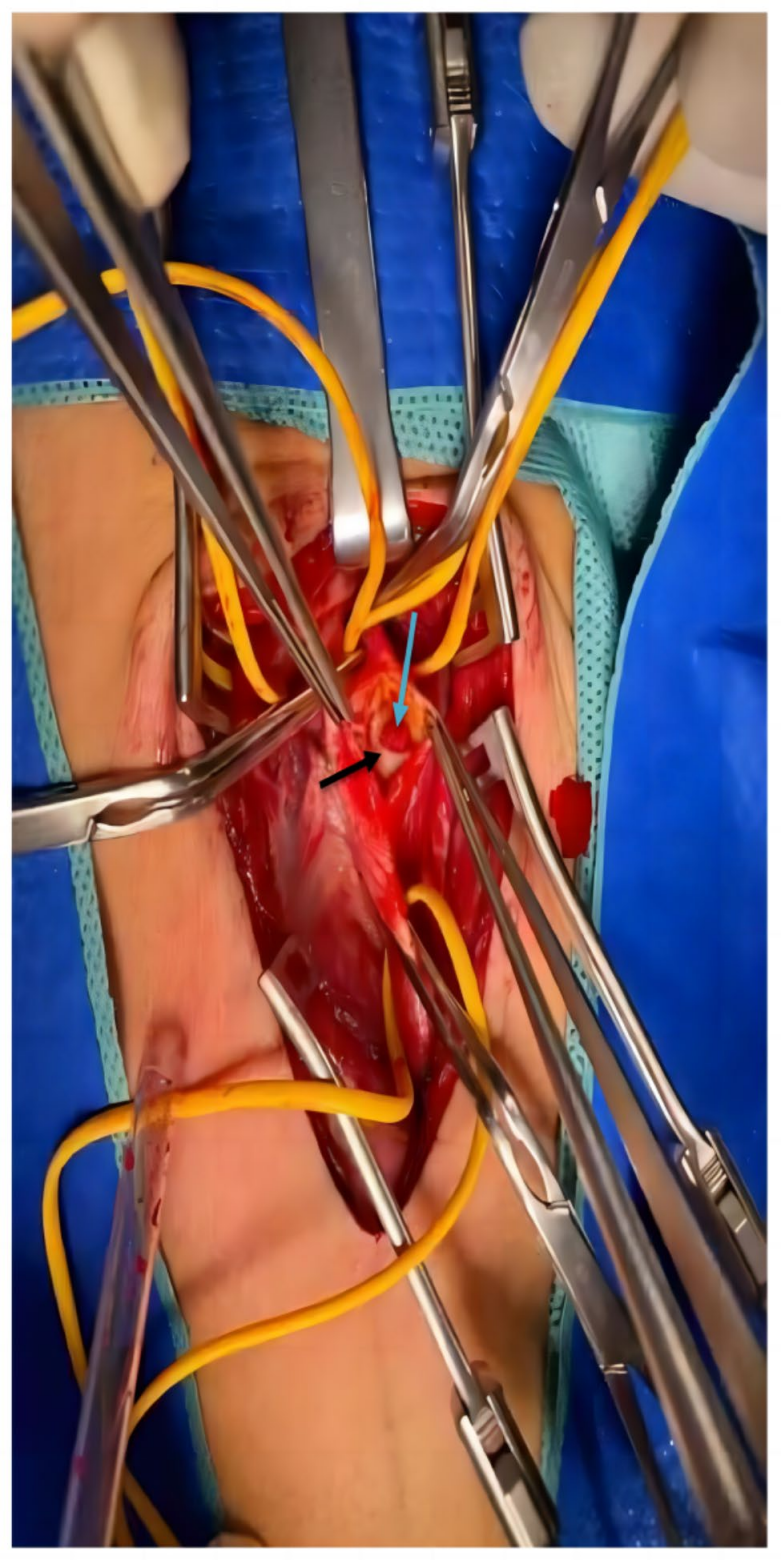
CaW (black arrow) and red thrombus (blue arrow) were observed during CEA

After 60 h of thrombolysis, carotid ultrasound found CaW in the posterior aspect of the left internal carotid bulb. Surprisingly, a slightly hyperecho mass of 6.1 × 3.9 mm was detected between web and posterior wall of the bulb, obvious eddy currents were detected around it. The CaW is a double layer of strong echo membranous structure protruding into the lumen, about 6.2 mm in length and 1.2 mm in thickness, consistent with the direction of blood flow. The angle between the web and the posterior wall was 35°. Left CaW with acute thrombosis in situ was highly suspected (Fig. 3). Emergent carotid endarterectomy (CEA) was performed under general anesthesia. During operation, blood clot was identified between the carotid intima and posterior arterial wall (Fig. 4). The thrombus was removed and endarterectomy was performed. Pathology revealed mixed thrombus, a small amount of fibrinoid nets, red blood cells and inflammatory cells. Pathology showed that the thickness of the wall was uneven, some of the wall tissues were hyalinized, and some of the wall tissues were mucoid. The patient recovered well and discharged on postoperative Day 10. The patient was followed up at 2 months, he was asymptomatic without evidence of recurrent cerebral infarction on carotid ultrasonography.

## Discussion

In this study, we reported a 49 years old male patient with recurrent cerebral infarction caused by CaW. Published studies reported that women and African Americans are significantly at high risk for CaW, especially in symptomatic CaW patients [6]. The patient with ischemic stroke caused by CaW usually has a younger age [7].A recently published epidemiological survey showed that the prevalence of CaW in transient ischemic attack/stroke, cryptogenic ischemic stroke, macrovascular occlusion stroke, carotid artery stenosis patients were 1.2%, 6.4%, 1.1%, and 4.4, respectively [8].The incidence of acute ischemic stroke (AIS) in patients diagnosed with CaW is as high as 89%, most of the AIS are ipsilateral, and the recurrence rate is also high [9].The incidence of CaW in young patients with cryptogenic stroke is as high as 25%, and most of the patient have proximal large vessel occlusion [6, 10]. The mechanism of stroke may be blood flow disorder, blood stasis, thrombosis, and embolism caused by abnormal CaW structure [4, 11]. Our patient is a typical cryptogenic ischemic stroke caused by ipsilateral carotid artery CaW, and the recurrent attack is probably due to the hemodynamic disorder caused by CaW, which leads to thrombosis formation and embolism to distal cerebral artery, resulting in cerebral infarction.

Previous reports have shown that CaW-induced embolic thrombosis of the great arteries is typical of mixed thrombosis [12].The pathological report of thrombosis in this patient was also consistent with this finding. Studies have found that there is obvious blood stasis and low wall shear stress around the CaW reticular structure, which is a hemodynamic risk factor for thrombosis [13].The artificial CaW model further confirmed that the lower the blood flow velocity, the more prone to turbulence. In particular, when the stenosis rate is higher and the angle is smaller, the turbulence intensity (TI) will be higher, which is also the pathogenesis of most CaW ischemic strokes [14]. In addition, the angle of CaW in stroke patients is smaller (mostly acute angle ≤ 90.1°), and with a significant longer length (≥ 3.1 mm) [15].The characteristic of this Caw on ultrasound (CaW length of 6.2 mm, with a small Angle of 35°), especially the obvious eddy around CaW detected by ultrasound supported this CaW was associated with a high-risk of stroke. In addition, the prolonged low basal blood pressure (about 100/70mmHg) leading to the insufficient carotid perfusion pressure in the acute phase of stroke maybe another important risk factor for the rapid recurrence of thrombosis.

At present, although the value of ultrasound alone in the diagnosis of CaW has been questioned, ultrasound still has irreplaceable advantages [16]. First of all, it is simple and convenient to operate, which can be evaluated and screened anytime and anywhere, and provide timely and effective guidance and suggestions for clinical practice. In this study, the new asymptomatic thrombosis can be timely and accurately detected, carotid ultrasound examination played a crucial role. Otherwise, this new thrombosis may cause another large artery embolic infarction, and the outcome is unpredictable. Secondly, ultrasound detection can quantitatively evaluate some basic morphological and hemodynamic characteristics of CaW, which is of more practical significance for stroke prevention. In addition, ultrasound examination technology is constantly innovating and developing. Recent studies have found that CaW display rate of CaW in longitudinal section of ultrasound is significantly higher than that in cross section, thus improving the diagnostic accuracy of CaW in ultrasound examination [17]. Furthermore, the slow flow sensitive ultrasonic microfluidic imaging (MFI) technology by duplex ultrasonography can significantly improve the diagnostic ability of CaW [18]. In the future, relevant clinical studies should be carried out to further verify the application value of carotid ultrasound in the prevention and control of long-term stroke for patients with CaW.

The treatment for CaW is still controversial. The conclusion of MR CLEAN trial has clearly warned that drug treatment alone may not be able to prevent the occurrence and recurrence of stroke caused by CaW [19]. For symptomatic patients with a carotid web in whom no other cause for stroke can be identified after detailed neurovascular work up, carotid endarterectomy or carotid artery stenting may be considered to prevent recurrent stroke [20]. At present, most studies tend to prefer CEA strategy for the prevention and control of the high recurrence rate of CaW stroke [21]. CEA may be appropriate as a treatment method along with prevention failure of antiplatelet agents in patients with CaW.

Although thrombosis caused by carotid web is rare, it has been reported by published study [12, 13, 22]. In this case, conservation antiplatelet therapy was not effective to prevent thrombosis and recurrence of stroke, and surgical intervention was indicated. CEA was arranged after thrombolysis when the patient’s condition was stable. Thrombosis was identified on duplex ultrasound after thrombolysis and emergent CEA was performed to prevent recurrent cerebral infarction. The patient recovered well.

## Conclusion

CaW is a rare cause of cerebral infarction. Routine duplex ultrasound examination is of great value for the diagnosis of CaW and prevention of stroke. Carotid endarterectomy is the treatment of choice in patients with CaW related thrombosis.

**Declarations**.

## Data Availability

All data are available in the manuscript.

## Abbreviations

CaW: Carotid web
CEA: Carotid endarterectomy
CAS: Carotid stenting
DSA: Digital subtraction angiography
CTA: Computed tomography angiography
CTP: CT perfusion
ICA: Internal carotid artery
MCA: Middle cerebral artery
MFI: Microfluidic imaging
AIS: Acute ischemic stroke

## References

[1] Hassani S, Nogueira RG, Al-Bayati AR, Kala S, Philbrook B, Haussen DC (2020). Carotid webs in Pediatric Acute ischemic stroke. J stroke Cerebrovasc diseases: official J Natl Stroke Association.

[2] Fu W, Crockett A, Low G, Patel V (2015). Internal carotid artery web: Doppler Ultrasound with CT Angiography correlation. J Radiol case Rep.

[3] Watanabe S, Tanaka K, Nakayama T, Kaneko M (1993). [Fibromuscular dysplasia at the internal carotid origin: a case of carotid web]. No shinkei geka Neurological surgery.

[4] Olindo S, Marnat G, Chausson N, Turpinat C, Smadja D, Gaillard N (2021). Carotid webs associated with ischemic stroke. Updated general review and research directions. Rev Neurol.

[5] Compagne KCJ, van Es A, Berkhemer OA, Borst J, Roos Y, van Oostenbrugge RJ (2018). Prevalence of carotid web in patients with Acute Intracranial Stroke due to Intracranial large vessel occlusion. Radiology.

[6] Mei J, Chen D, Esenwa C, Gold M, Burns J, Zampolin R (2021). Carotid web prevalence in a large hospital-based cohort and its association with ischemic stroke. Clin Anat (New York NY).

[7] Yang T, Yoshida K, Maki T, Fushimi Y, Yamada K, Okawa M et al. Prevalence and site of predilection of carotid webs focusing on symptomatic and asymptomatic japanese patients. J Neurosurg. 2021:1–7; doi: 10.3171/2020.8.jns201727. 10.3171/2020.8.JNS201727 33668027

[8] Zhang J, Yan Y, Yao W, Liu J, Cui L. Multimodality imaging of carotid web: a case report and literature review. Vascular. 2022;17085381221084809. 10.1177/17085381221084809. 10.1177/17085381221084809 35306924

[9] Sajedi P, Chelala L, Nunez-Gonalez J, Cronin C, Kittner S, Zhuo J (2019). Carotid webs and ischemic stroke: experiences in a comprehensive stroke center. J neuroradiology = Journal de neuroradiologie.

[10] Turpinat C, Collemiche FL, Arquizan C, Molinari N, Cagnazzo F, Mourand I (2021). Prevalence of carotid web in a french cohort of cryptogenic stroke. J Neurol Sci.

[11] Labeyrie MA, Serrano F, Civelli V, Jourdaine C, Reiner P, Saint-Maurice JP (2021). Carotid artery webs in embolic stroke of undetermined source with large intracranial vessel occlusion. Int J stroke: official J Int Stroke Soc.

[12] Gao Q, Hu S, Yang X, Wang J, Lu J, Wang D. Histologic differences between in situ and embolized carotid web thrombi: a case report. BMC Neurol. 2021;21(1:398). 10.1186/s12883-021-02428-w. 10.1186/s12883-021-02428-w PMC8513242 34645398

[13] Ozaki D, Endo T, Suzuki H, Sugiyama SI, Endo K, Itabashi R (2020). Carotid web leads to new thrombus formation: computational fluid dynamic analysis coupled with histological evidence. Acta Neurochir.

[14] Bae T, Ko JH, Chung J (2021). Turbulence intensity as an Indicator for ischemic stroke in the carotid web. World Neurosurg.

[15] Tabibian BE, Parr M, Salehani A, Mahavadi A, Rahm S, Kaur M, et al. Morphological characteristics of symptomatic and asymptomatic carotid webs. J Neurosurg. 2022;1–6. 10.3171/2022.2.jns212310. 10.3171/2022.2.JNS212310 35426815

[16] Hassani S, Nogueira RG, Al-Bayati AR, Sachdeva R, McDaniel M, Haussen DC (2020). Intravascular Ultrasound in Carotid web. J neurointerventional Surg.

[17] Ben Z, Wang J, Zhan J, Chen S. Ultrasonic characteristics of carotid webs. Neuroradiology. 2022;64. 10.1007/s00234-021-02757-0. 1:95 – 8; doi:. 10.1007/s00234-021-02757-0 34251500

[18] Fontaine L, Guidolin B, Viguier A, Gollion C, Barbieux M, Larrue V (2022). Ultrasound characteristics of carotid web. J neuroimaging: official J Am Soc Neuroimaging.

[19] Guglielmi V, Compagne KCJ, Sarrami AH, Sluis WM, van den Berg LA, van der Sluijs PM (2021). Assessment of recurrent stroke risk in patients with a carotid web. JAMA Neurol.

[20] Naylor R, Rantner B, Ancetti S, de Borst GJ, De Carlo M, Halliday A, Kakkos SK, Markus HS, McCabe DJH, Sillesen H, van den Berg JC, Vega de Ceniga M, Venermo MA, Vermassen FEG, Esvs Guidelines Committee, Antoniou GA, Bastos Goncalves F, Bjorck M, Chakfe N, Coscas R, Dias NV, Dick F, Hinchliffe RJ, Kolh P, Koncar IB, Lindholt JS, Mees BME, Resch TA, Trimarchi S, Tulamo R, Twine CP, Wanhainen A, Reviewers D, Bellmunt-Montoya S, Bulbulia R, Darling RC 3rd, Eckstein HH, Giannoukas A, Koelemay MJW, Lindström D, Schermerhorn M, Stone DH, editors. ‘s Choice - European Society for Vascular Surgery (ESVS) 2023 Clinical Practice Guidelines on the Management of Atherosclerotic Carotid and Vertebral Artery Disease. Eur J Vasc Endovasc Surg. 2023 Jan;65(1):7-111. doi: 10.1016/j.ejvs.2022.04.011. Epub 2022 May 20. PMID: 35598721. 10.1016/j.ejvs.2022.04.011 35598721

[21] Haynes J, Raz E, Tanweer O, Shapiro M, Esparza R, Zagzag D et al. Endarterectomy for symptomatic internal carotid artery web. J Neurosurg. 2020:1–8; doi: 10.3171/2020.5.jns201107. 10.3171/2020.5.JNS201107 32858515

[22] Al-Dulaimi MW, Ridha M, Small JE, Tilem M, Voetsch B, Helenius J. Acute thrombosis on a carotid web associated with an ipsilateral embolic stroke. Neurol 2020 Nov 17;95(20):931–2. doi: 10.1212/WNL.0000000000010972. Epub 2020 Oct 1. PMID: 33004604. 10.1212/WNL.0000000000010972 33004604

